# Loss of Leucine-Rich Repeat Kinase 2 (LRRK2) in Rats Leads to Progressive Abnormal Phenotypes in Peripheral Organs

**DOI:** 10.1371/journal.pone.0080705

**Published:** 2013-11-14

**Authors:** Marco A. S. Baptista, Kuldip D. Dave, Mark A. Frasier, Todd B. Sherer, Melanie Greeley, Melissa J. Beck, Julie S. Varsho, George A. Parker, Cindy Moore, Madeline J. Churchill, Charles K. Meshul, Brian K. Fiske

**Affiliations:** 1 Research Programs, The Michael J. Fox Foundation for Parkinson’s Research, New York, New York, United States of America; 2 WIL Research, Ashland, Ohio, United States of America; 3 Research Services, VA Medical Center/Portland and the Department of Behavioral Neuroscience and Pathology, Oregon Health and Science University, Portland, Oregon, United States of America; Ecole Polytechnique Federale de Lausanne (EPFL), Switzerland

## Abstract

The objective of this study was to evaluate the pathology time course of the LRRK2 knockout rat model of Parkinson’s disease at 1-, 2-, 4-, 8-, 12-, and 16-months of age. The evaluation consisted of histopathology and ultrastructure examination of selected organs, including the kidneys, lungs, spleen, heart, and liver, as well as hematology, serum, and urine analysis. The LRRK2 knockout rat, starting at 2-months of age, displayed abnormal kidney staining patterns and/or morphologic changes that were associated with higher serum phosphorous, creatinine, cholesterol, and sorbitol dehydrogenase, and lower serum sodium and chloride compared to the LRRK2 wild-type rat. Urinalysis indicated pronounced changes in LRRK2 knockout rats in urine specific gravity, total volume, urine potassium, creatinine, sodium, and chloride that started as early as 1- to 2-months of age. Electron microscopy of 16-month old LRRK2 knockout rats displayed an abnormal kidney, lung, and liver phenotype.  In contrast, there were equivocal or no differences in the heart and spleen of LRRK2 wild-type and knockout rats. These findings partially replicate data from a recent study in 4-month old LRRK2 knockout rats [1] and expand the analysis to demonstrate that the renal and possibly lung and liver abnormalities progress with age. The characterization of LRRK2 knockout rats may prove to be extremely valuable in understanding potential safety liabilities of LRRK2 kinase inhibitor therapeutics for treating Parkinson’s disease.

## Introduction

Parkinson’s disease (PD) is the second most common neurodegenerative disease, affecting 1-2% of the population over the age of 60 [2,3]. The cardinal clinical features include tremor, rigidity, bradykinesia and/or postural instability, as well as neuropathological loss of dopaminergic neurons in the substantia nigra (SN), decreased dopamine (DA) neurotransmission, and the presence of neuronal intracellular Lewy body (LB) inclusions [2]. In addition, non-motor features such as depression, constipation, pain, and sleep disorders are important manifestations of the disease [4].

Historically believed to have no strong genetic component, genetic mutation or variation in a number of genes is now recognized as causal or risk-associated factors involved in a growing number of PD cases [5–7]. Mutations in the leucine-rich repeat kinase 2 (LRRK2) gene are the most common cause of familial and late-onset PD identified to date [3]. The most common LRRK2 mutation, G2019S, accounts for as much as 30-40% of Parkinsonism in Ashkenazi Jews and North African Arab-Berber populations [8,9]. Furthermore, LRRK2 mutations account for up to 2% of sporadic Parkinsonism [10]. The LRRK2 gene encodes a large multi-domain protein containing an ankyrin repeat region, a leucine-rich repeat domain, a Ras of complex protein GTPase domain, a C-terminal of Roc domain, a kinase domain, and a WD40 domain [11]. The LRRK2 G2019S mutation in the kinase domain appears to increase its enzymatic activity [12] and since LRRK2-related PD and sporadic PD display a similar phenotype [13], pharmaceutical companies are pursuing LRRK2 kinase inhibitors to reduce this gain-of-function as a promising therapeutic option for people with PD. 

To be viable for human therapeutic development, drug makers must demonstrate that inhibition of LRRK2 activity is safe. In the absence of optimal tool compounds (i.e., potent and selective to LRRK2), researchers have utilized genetically modified rodent models to explore potential liabilities of targeting LRRK2 kinase activity. Studies in LRRK2-deficient mice have found morphological and histopathological abnormalities in both kidney and lung tissue that have been associated with impairments in the autophagy pathway [14–17]. LRRK2 knockout (KO) mice display large kidneys that are dark red with microscopic presence of microvacuoles in the proximal tubule epithelial cells. A lung phenotype (increased number and size of lamellar bodies) has also been found in LRRK2 KO but not kinase-dead (KD) mice, suggesting that the LRRK2 protein-protein binding domains, rather than the kinase domain, may be crucial for normal lung function [16]. However, the LRRK2 mouse studies that have been published to date have not examined any clinical chemistry or other biomarkers that may be associated with these deficits This information could be critical to guiding the development of appropriate safety measures for future clinical trials. 

Recently, it was reported that LRRK2 KO rats at 4-months of age exhibit perturbations in renal morphology accompanied by significant decreases of lipocalin-2 (NGAL) in both urine and plasma [1]. Although consistent with reports in KO mice, this finding is inconsistent with renal damage since an increase in NGAL is an early responder of nephrotoxicity and tubular damage. The authors speculate that the decrease in NGAL may be independent of renal function but associated with alterations of immune homeostasis [1]. Furthermore, significant alterations in the cellular composition of the spleen between LRRK2 KO rats and wild-type (WT) animals were detected with subtle differences in response to dual infection with rat-adapted influenza virus and *Streptococcus pneumoniae*. A molecular pathway analysis of LRRK2 revealed links between LRRK2 and the thioredoxin system, which interacts with PRDX3, TXNIP, and TXNRD1. These proteins are associated with nutrient sensing, adiposity, and human obesity. The authors suggest that there might be a link between the reported LRRK2 KO weight gain, LRRK2 deficiency, and the thioredoxin pathway [1]. 

Given that this characterization of LRRK2 KO rats was limited to one age and there is still ambiguity regarding the clinical pathology markers associated with the LRRK2 KO renal phenotype, the present study extends these findings by examining the morphology, histopathology, ultrastructure, blood, and urine chemistry in LRRK2 KO and WT rats in 6 different age groups spanning a 16-month period.

## Materials and Methods

Ethics Statement: All animal work in these studies is in compliance with the National Institutes of Health for humane animal welfare and has been approved by WIL Research and VA Medical Center/Portland IACUC committees.

### LRRK2 KO and Long-Evans WT rats

Three separate cohorts of homozygous LRRK2 KO and WT male Long Evans rats from Sigma Advance Genetic Engineering (SAGE) Laboratories were maintained and aged to 1-, 2-, 4-, 8-, 12-, and/or 16-months of age. All breeding was conducted as homozygous x homozygous, so that the WT and KO rats were not littermates.

For the first cohort (4-, 8-, and 12-months of age; n=4 per group), organs were examined macroscopically and weighed. Rats were euthanized by decapitation and tissues were snap frozen in liquid nitrogen.

For the second cohort (1-, 2-, and 8-months of age; n=4 per group), rats were deeply anesthetized by an intraperitoneal injection of sodium pentobarbital and perfused *in situ* (4.0% paraformaldehyde in a 0.1 M phosphate buffer solution). Tissues were dissected and placed in 10% neutral-buffered formalin for 24‑48 hours and then transferred to 70% ethanol. At the time of necropsy, the tissues were collected and placed in 10% neutral-buffered formalin fixative. 

For the third cohort, 16-month old rats (n=4 per group) were anesthetized and then perfused transcardially with 350 ml of electron microscopy (EM) fixative, consisting of 1% glutaraldehyde, 0.5% paraformaldehyde, and 0.1% picric acid in 0.1 M phosphate buffer. The different tissue preparations for the three cohorts arose due to the varying requirements of analyzing the samples.

### Microscopic Examination (Cohorts 1 and 2)

Microscopic examination of hematoxylin-eosin (H&E) stained paraffin sections was performed on all tissues collected at necropsy from all animals. Also, since LRRK2 has a role in autophagy and the kidney has been shown to be affected in KO rodents, tubular lysosomes were assessed using a variety of histochemical and immunohistochemical methods (see Supplement S1 for specific methodologies). Stained histologic sections were examined by light microscopy. Grading of lesions noted on H&E stained sections and staining patterns in histochemical and immunohistochemical stained sections is detailed in Supplement S2.

#### Lipofuscin stain (AFIP method; kidney, lung, spleen, heart, and liver)

This histochemical stain using carbol fuchsin and picric acid detects residues of lysosomal digestion. Lipofuscin is considered a pigment associated with cell organelle damage and aging [18]. 

#### Chromotrope aniline blue (CAB; kidney only)

This stain is used to detect protein containing hyaline droplets in the tubular epithelium – the CAB has a high affinity for protein and stains it a bright red [19].

#### LAMP-1 and LAMP-2 IHC (Kidney, Lung, Spleen, Heart, and Liver)

These markers are also known as CD107A (-1) and CD107B (-2) which are lysosomal membrane proteins (late and early endosomes, respectively) that are translocated to the cell surface after activation [20]. 

#### N-acetylglucosaminidase-IHC (NAGLU; kidney only)

This is a lysosomal enzyme that is involved in the breakdown of glycosaminoglycan [21].

#### Kidney Injury Molecule-1 IHC (KIM-1; kidney only)

This protein is expressed in low levels in a normal kidney and is a type 1 cell membrane glycoprotein which regulates cell-cell adhesion and endocytosis. Endocytosis is one function of the proximal tubular epithelium where lysosomes play a crucial role [22].  

### Electron Microscopy (Cohort 3)

Following perfusion of cohort 3, lung, liver, kidney, spleen, heart and brain were collected and placed in EM fix overnight at 4°C. Each organ was then cut into 2 mm^3^ sections, EM processed using a newly developed microwave (Pelco BioWave, Ted Pella, Inc.) procedure as previously described [23], and embedded in Epon-Spurs resin overnight at 60° C. After each tissue block was evaluated for quality selected blocks were thin sectioned on the ultramicrotome to 60 nm in thickness using a diamond knife (Diatome, Hatfield, PA) and then counterstained with uranyl acetate and lead citrate. Images were then taken randomly throughout the tissue section with a JEOL 1400 Transmission Electron Microscope (JEOL, Peabody, MA) and photographed using a digital camera (AMT, Danvers, MA). Between 30 and 50 photos per section of tissue were taken. Once morphological changes between the LRRK2 KO and the WT were found, further analysis was performed, using ImagePro Plus software (Media Cybernetics, Rockville, MD). In the lung the number of lamellar bodies per cell, the area of the lamellar bodies, and the area of Alveolar Type II cells were calculated. In the liver the area of the hepatic cell and lipid droplets were calculated as well as the number of lipid droplets per cell. After data were collected, differences between the LRRK2 KO and WT groups were determined using the Students’ *t*-test. Data were then graphed using Graphpad Prism. 

### Clinical Pathology

Hematology, coagulation, serum chemistry, and urinalysis parameters were evaluated on all animals in cohort 2 (1-, 2-, and 8-months old) just prior to the scheduled necropsy. Animals that were at least 2-months of age were fasted overnight prior to blood collection. Blood samples were collected via the jugular vein. Urine was collected overnight using metabolism cages. Anticoagulants used were potassium EDTA for hematology parameters and sodium citrate for coagulation parameters. Anticoagulants were not used for serum chemistry parameters. Clinical pathology parameters evaluated are listed in Supplement S3. Urine chemistry sodium, potassium, and chloride were measured (mEq/L) and normalized to urine creatinine (mg/L).

### Statistical analysis

Organ weights (absolute, relative to body, and relative to brain weights) and clinical pathology parameters were analyzed by a two-sample *t*-test.

## Results

### Gross Observations and Organ Weights

All animals were apparently healthy, viable, and survived to the scheduled necropsy (referred to as day 0). Mean body weights in the LRRK2 KO group were higher than the Long Evans WT group at all ages on study days -1 and 0; the differences were significant (all statistical analyses employed two-tailed *t*-tests) (p<0.05 or p<0.01) at 2- and 8-months of age. Absolute brain weight, brain length, and brain width values were higher in LRRK2 KO rats in all age groups and all of these differences from the control group were statistically significant (p<0.01) except for the brain length value at 1-month of age. Brain measurement changes were not associated with microscopic findings. 

#### Histopathological Observations

**Figure 1:**
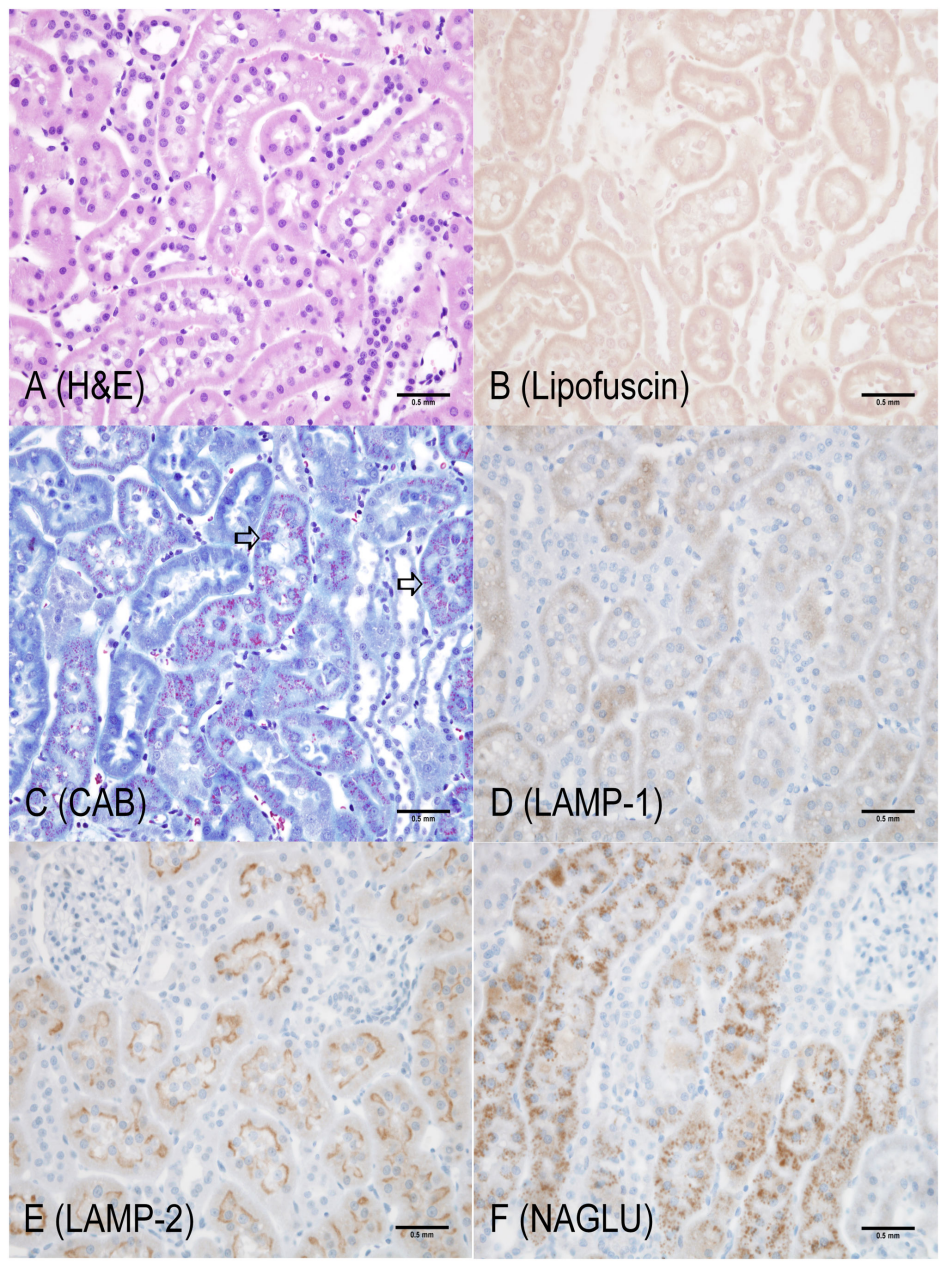
4-month old Long Evans WT rat (Cortex, kidney). Representative of Long Evans WT kidney. Similarities or differences in KO rats were compared to concurrent age WT controls. **A**: Hematoxylin and Eosin – no observable abnormalities; **B**: Lipofuscin (AFIP method) – lipofuscin positive material not observed; **C**: Chromotrope Aniline Blue – fine granular cytoplasmic staining (red) indicating small amounts of protein in lysosomes of proximal tubular epithelium (open arrow); **D**: LAMP-1 – diffuse, cytoplasmic, fine granular staining in proximal tubular epithelium; **E**: LAMP-2 – intense staining of apical cytoplasm of proximal tubular epithelium; **F**: NAGLU – fine granular cytoplasmic staining of proximal tubular epithelium. Scale bar = 0.5 mm

**Figure 2:**
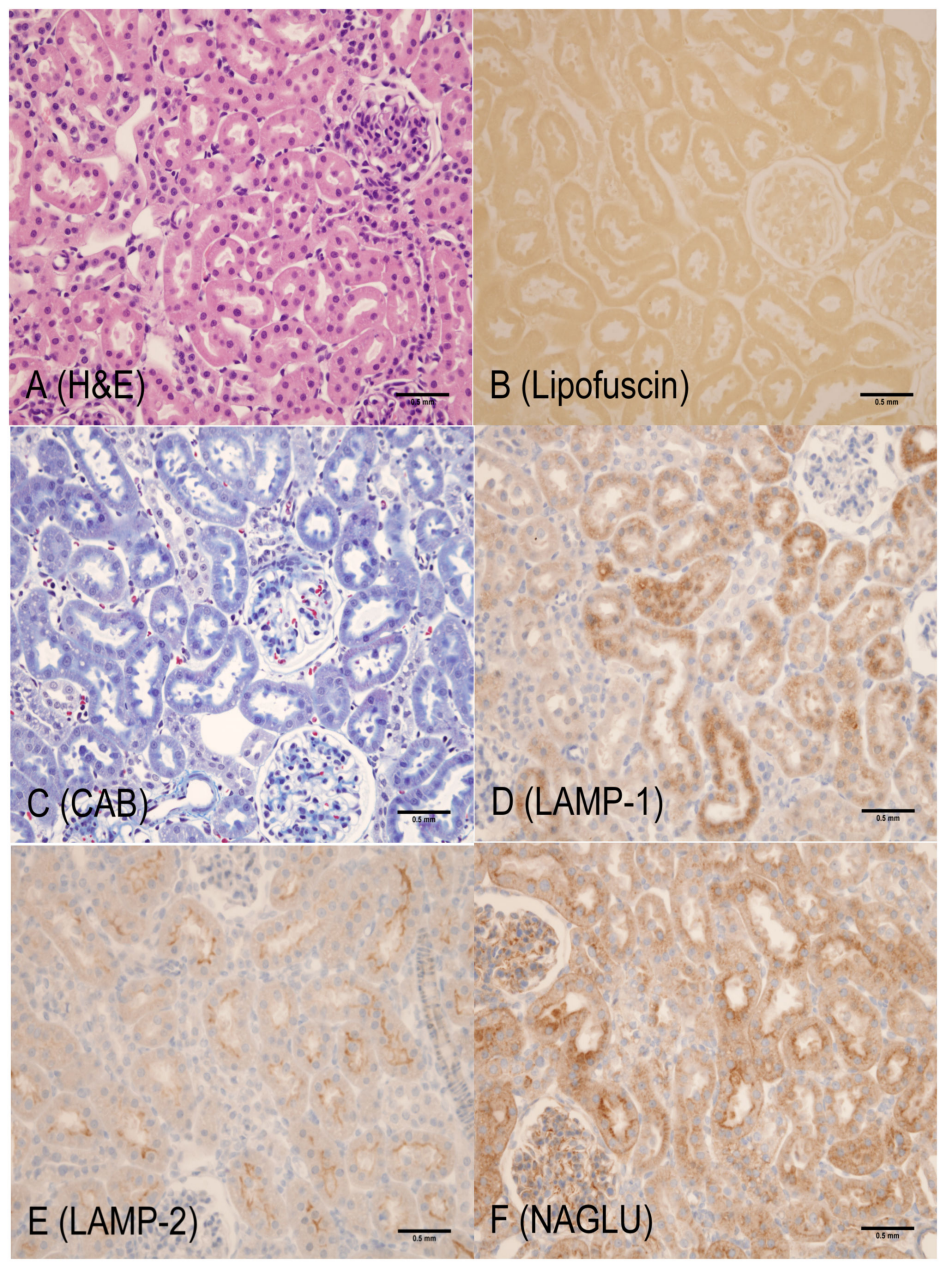
1-month old LRRK2 KO rat (Cortex, kidney). A: Hematoxylin and Eosin – no observable abnormalities; B: Lipofuscin (AFIP method) – lipofuscin positive material not observed; C: Chromotrope Aniline Blue – CAB positive staining not readily visible and similar to WT rat; D: LAMP-1 – diffuse, cytoplasmic, fine granular staining with a slight increase in intensity in proximal tubular epithelium; E: LAMP-2 - intense staining of apical cytoplasm in proximal tubular epithelium similar to WT control; F: NAGLU – fine, diffuse, granular cytoplasmic staining and apical staining in proximal tubular epithelium similar to WT control (the 1, 2 and 8 month old WT rats in the second cohort had similar baseline NAGLU staining that was slightly different (apical and diffuse cytoplasmic) from those rats in the first cohort (diffuse cytoplasmic). Scale bar = 0.5 mm

**Figure 3:**
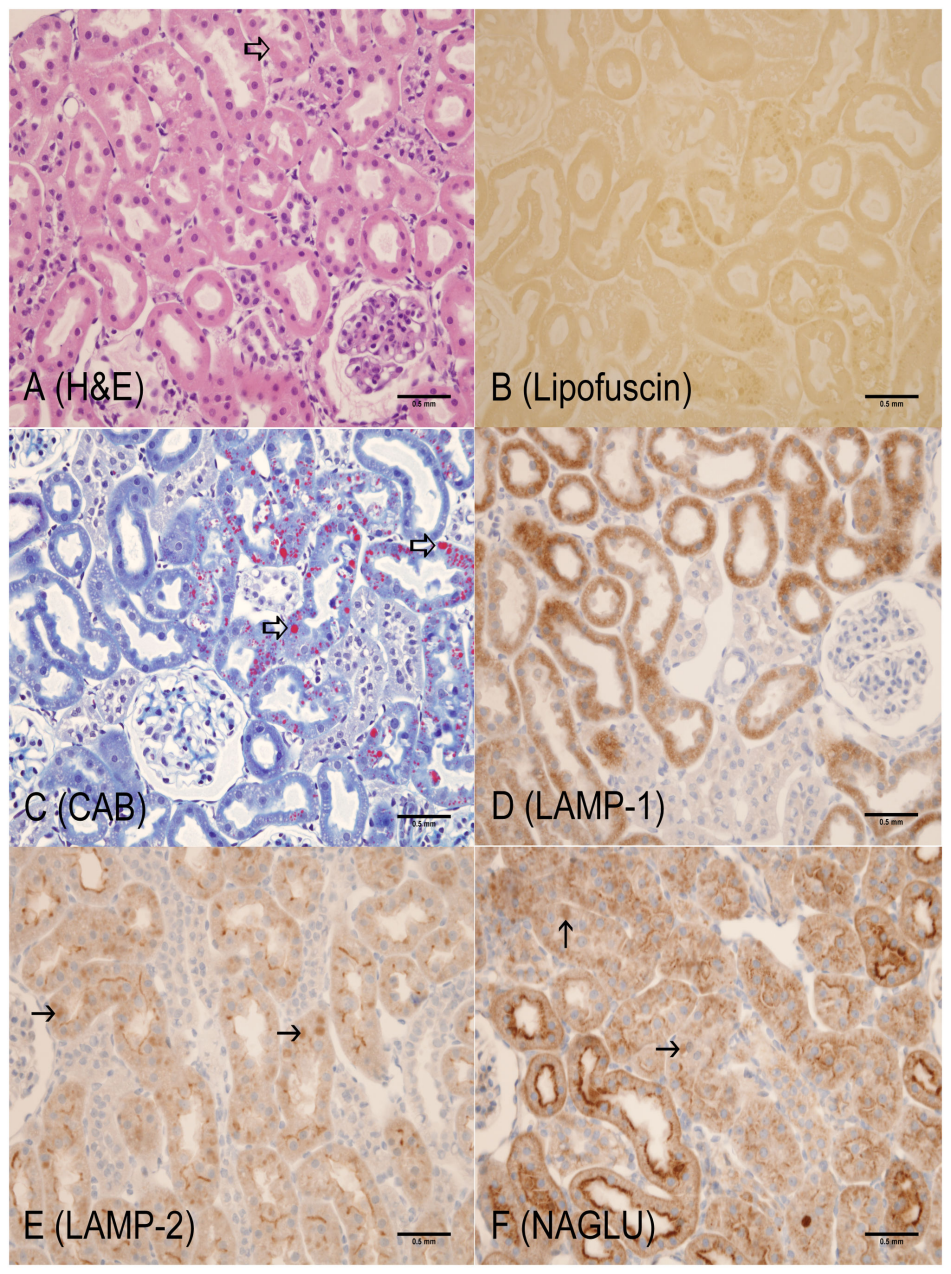
2-month old LRRK2 KO rat (Cortex, kidney). A: Hematoxylin and Eosin – slight increase in size and variability of hyaline droplets in proximal tubular epithelium (open arrow); B: Lipofuscin (AFIP method) – Lipofuscin positive material not observed; C: Chromotrope Aniline Blue – increased size, number and variability of hyaline droplets in proximal tubular epithelium (open arrows). Hyaline droplet variability was more easily identified using CAB staining when compared to H&E stains; D: LAMP-1 – increased cytoplasmic staining intensity in proximal tubular epithelium; E: LAMP-2 – increased globular cytoplasmic staining (solid arrows) with apical cytoplasmic staining- persisting in proximal tubular epithelium; F: NAGLU – increased globular staining in proximal tubular epithelium (solid arrow). Scale bar = 0.5 mm

**Figure 4:**
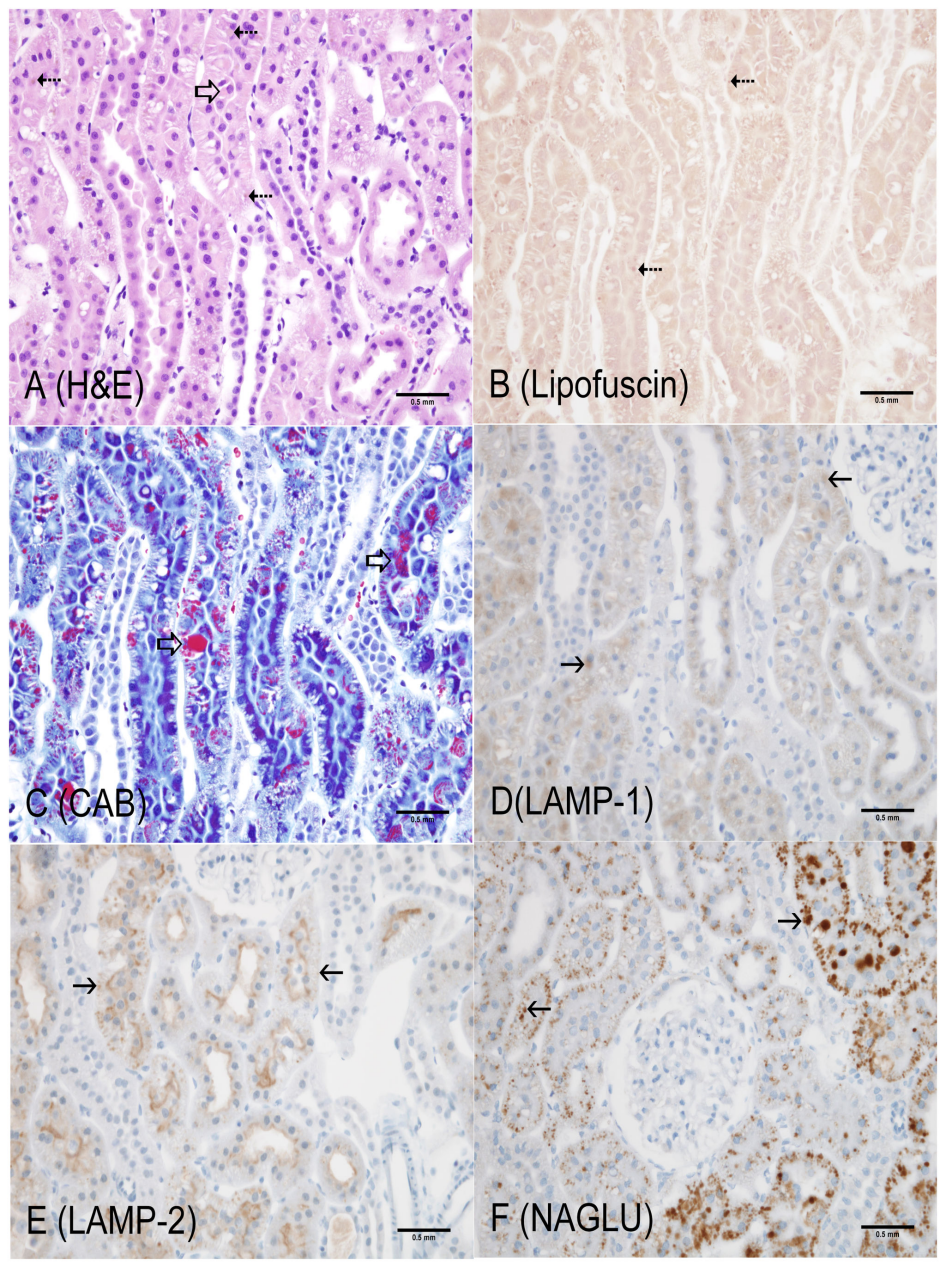
4-month old LRRK2 KO rat (Cortex, kidney). A: Hematoxylin and Eosin – brown, globular pigment (dotted arrows) and intracytoplasmic, clear vacuoles in proximal tubular epithelium with occasional hyaline droplets (open arrow); B: Lipofuscin (AFIP method) – positive dark red staining of pigment (dotted arrows) and clear vacuoles in proximal tubular epithelium; C: Chromotrope Aniline Blue – Increase in number, size and variability of hyaline droplets (red, open arrows) and clear cytoplasmic vacuoles in proximal tubular epithelium; D: LAMP-1 – Globular cytoplasmic staining in proximal tubular epithelium (solid arrows); E: LAMP-2 – increased globular cytoplasmic staining (solid arrows) with apical staining persisting in proximal tubular epithelium; F: NAGLU – increase intensity of globular cytoplasmic staining (open arrows). Scale bar = 0.5 mm

**Figure 5:**
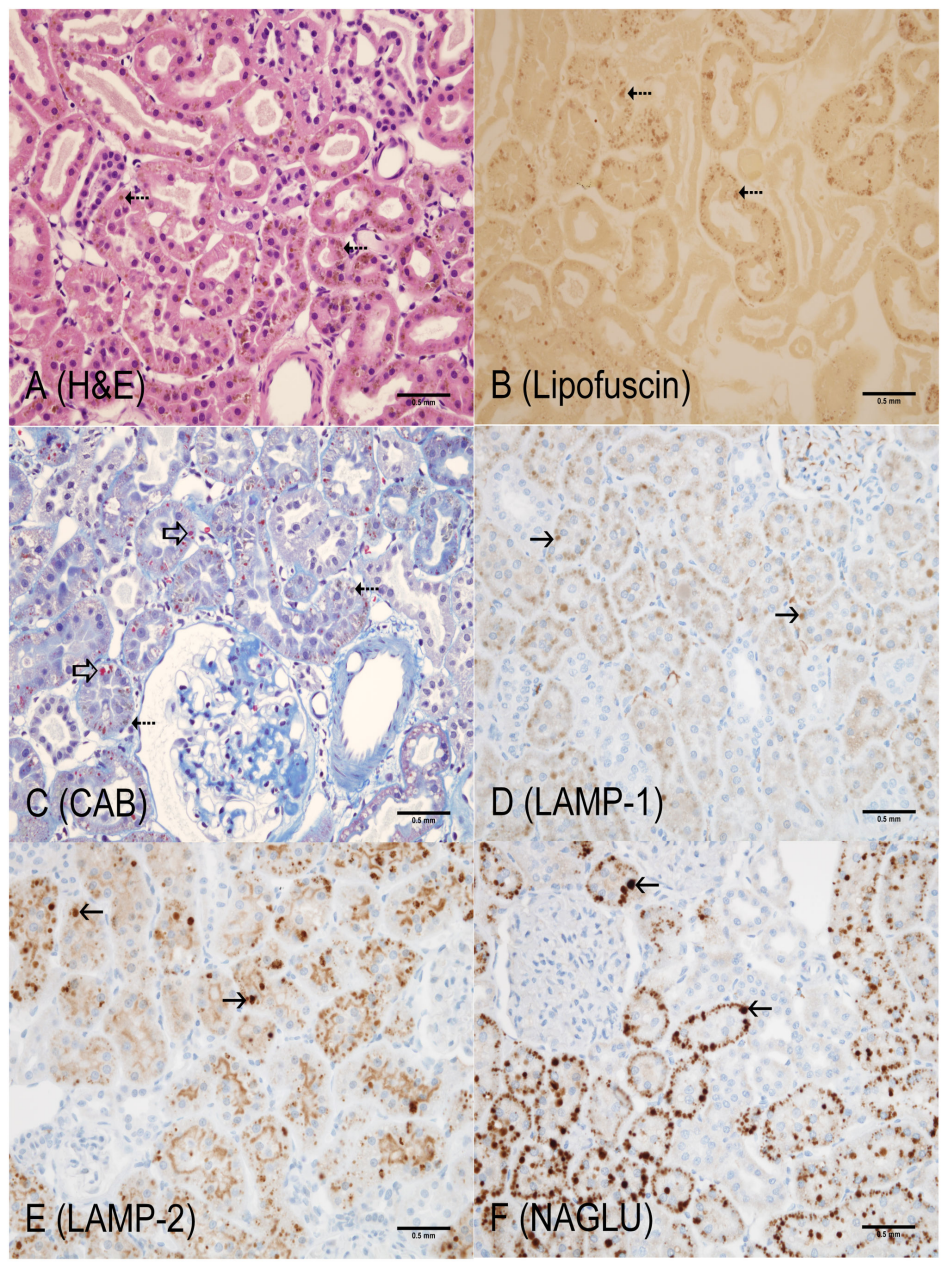
8-month old LRRK2 KO rat (Cortex, kidney). A: Hematoxylin and Eosin – increased amounts of brown pigment (dotted arrows) with cytoplasmic vacuolation in proximal tubular epithelium; B: Lipofuscin (AFIP method) – positive dark red staining of pigment in proximal tubular epithelium (dotted arrows); C: Chromotrope Aniline Blue – hyaline droplets (red open arrows) are smaller and not as variably-sized. Hyaline droplets are occasionally noted along with brown pigment in proximal tubular epithelium; D: LAMP-1 – Increased globular cytoplasmic staining (solid arrows) in proximal tubular epithelium; E: LAMP-2 – increased globular cytoplasmic staining with increased intensity (solid arrow) and persistence of apical cytoplasmic staining; F: NAGLU – globular cytoplasmic staining is increased in intensity in proximal tubular epithelium (solid arrows). Scale bar = 0.5 mm

**Figure 6:**
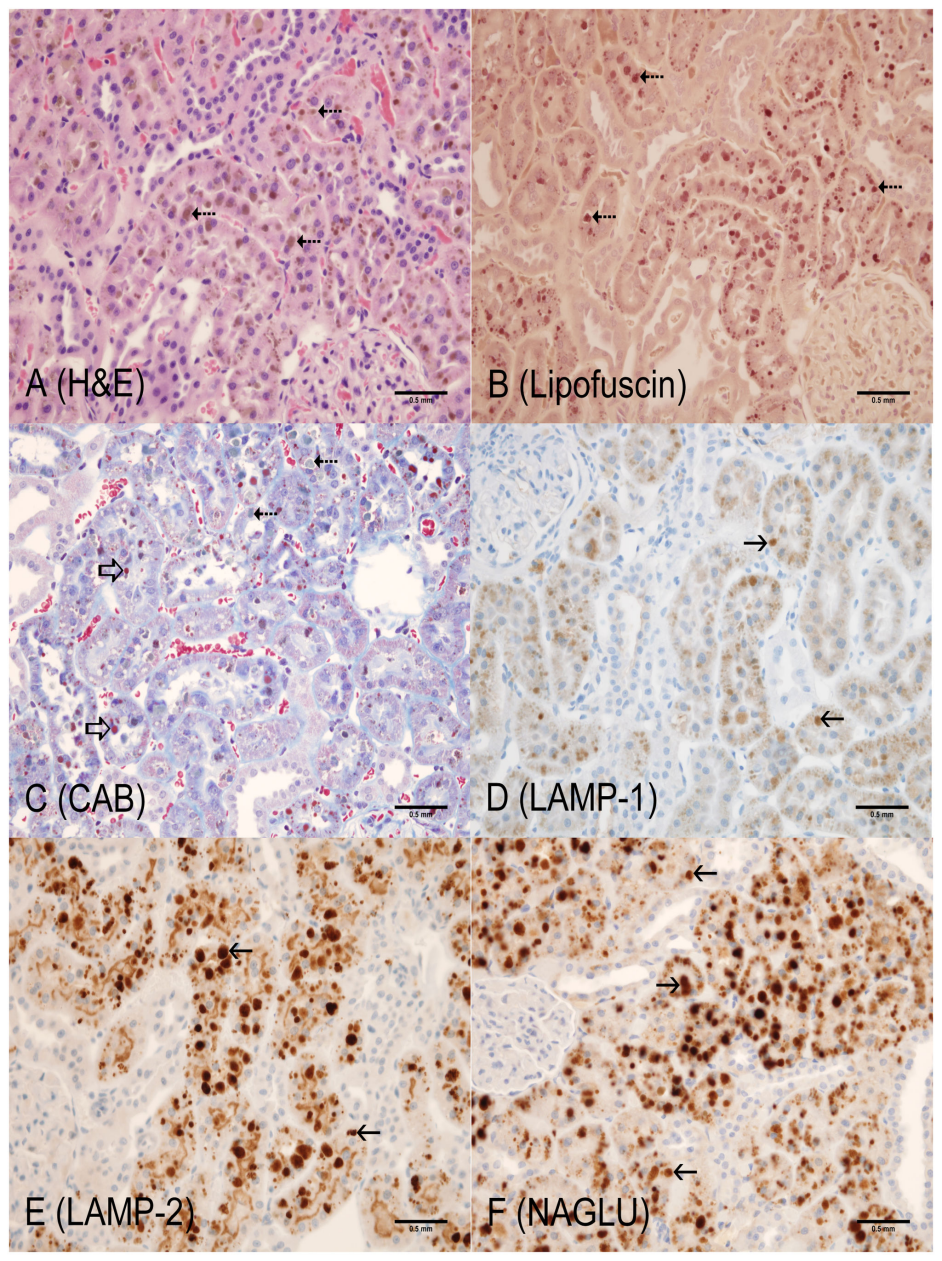
12-month old LRRK2 KO rat (Cortex, kidney). A: Hematoxylin and Eosin – increased amounts of brown pigment (dotted arrows) in proximal tubular epithelium; B: Lipofuscin (AFIP method) – positive dark red staining of pigment (dotted arrows); C: Chromotrope Aniline Blue – positive red hyaline droplets are small and not as variable (open arrows) and are observed in proximal tubular mostly separate from brown pigment (dotted arrows); D: LAMP-1 – increased globular cytoplasmic staining (solid arrows); E: LAMP-2 – increased globular cytoplasmic staining with increased intensity (solid arrows) and persistence of apical cytoplasmic staining; F: NAGLU – increase in intensity of globular cytoplasmic staining (solid arrows). Scale bar = 0.5 mm

The most unequivocal morphologic phenotype associated with knockout of the LRRK2 gene was observed in the kidney. Kidney changes manifested grossly as dark red kidneys in 8- and 12- month old rats and microscopically as hyaline droplets, cytoplasmic vacuolation, and brown pigment accumulation in renal tubules of 2-, 4-, 8-, and 12-month old rats (Table 1). No LRRK2 KO-related microscopic changes were noted at 1-month of age. Microscopic changes observed in the kidney with the histochemical and immunohistochemical stains are detailed below and are shown in Figures 1 through 7. Microscopic observations in the Long Evans WT rats (histochemical and immunohistochemical) were generally similar amongst the 1-, 2-, 4-, 8-, and 12-month age groups with the exception of chronic progressive nephropathy incidence and severity. Figure 1 details microscopic findings of the 4-month Long Evans WT age group, which was considered generally representative of all WT age groups. Figures 2, 3, 4, 5, and 6 show the microscopic findings in the LRRK2 KO rats at 1-, 2-, 4-, 8-, and 12-months of age, respectively.

**Table 1:** Summary of histochemical and immunohistochemical stain results for 4-month WT, 1-, 2-, 4-, 8-, and 12-month LRRK2 KO rats.

**Observation (Figure panel)**	**4-mo. WT ** Figure **1**	**1-mo. Lrrk2 KO ** Figure **2**	**2-mo. Lrrk2 KO ** Figure **3**	**4-mo. Lrrk2 KO ** Figure **4**	**8-mo. Lrrk2 KO ** Figures **5** & **7**	**12-mo. Lrrk2 KO ** Figure **6**	**8-mo. WT ** Figure **7**
**Brown pigment**	-	-	-	++	+++	++++	NA
H&E (A)	-	-	-	+	+++	++++	NA
Lipofuscin (B)	-	-	-	++	+++	++++	NA
**Hyaline droplets**	+	-	++	+++	++	++	
H&E (A, not as easily identified with this stain)	-	-	+	+	-	-	NA
CAB (C)	+	-	++	+++	++	++	NA
**Lysosomes**	+	+	++	++	+++	++++	NA
LAMP-1 (D)	+	+	++	++	+++	+++	NA
LAMP-2 (E)	+	+	++	++	+++	++++	NA
NAGLU (F)	+	+	++	++	+++	++++	NA
**KIM-1**	NA	NA	NA	NA	+++ (KO)	NA	+ (WT)

**Figure 7:**
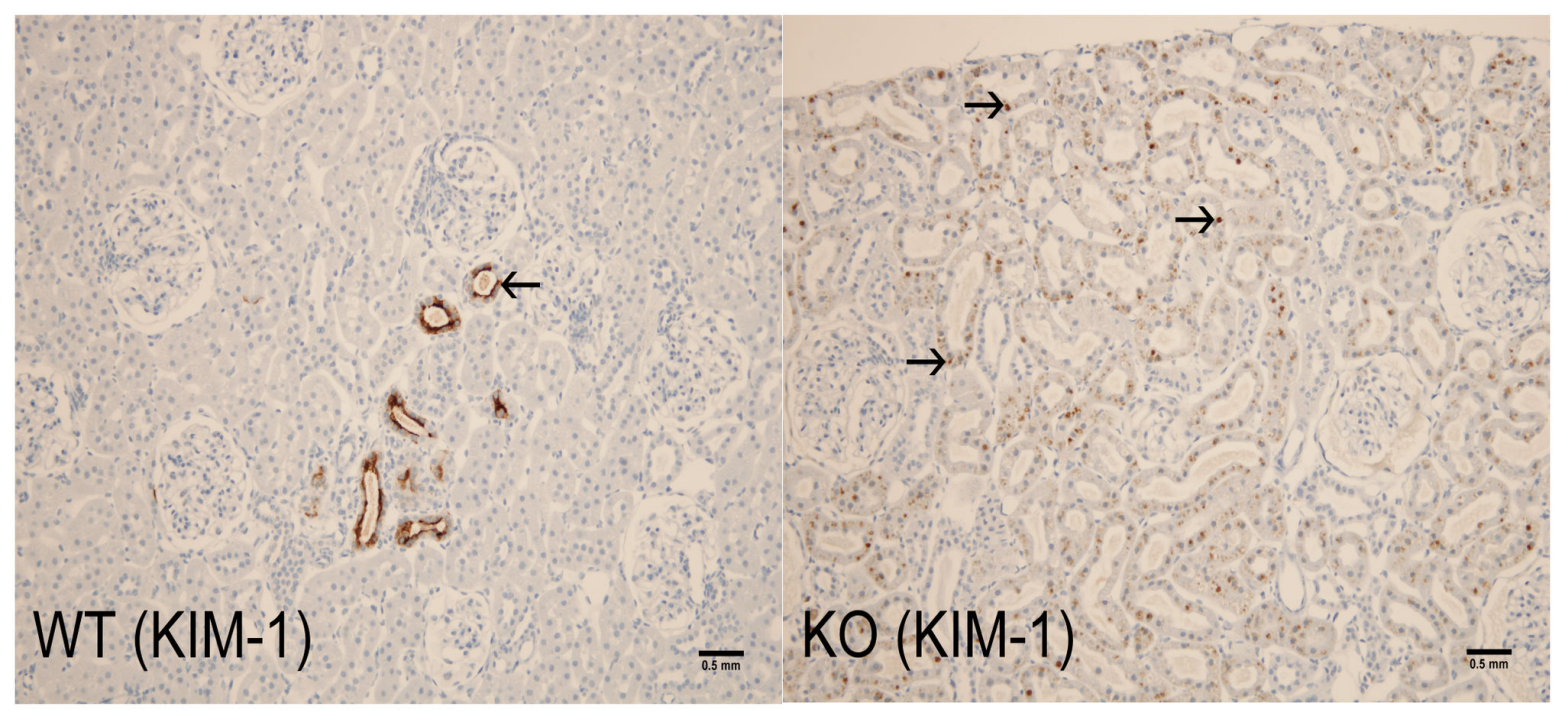
8-month old LRRK2 KO rat (Cortex, kidney). WT: Kidney Injury Molecule-1 (KIM-1) staining in the Long Evans WT rat. There is focal positive staining (solid arrows) within tubular epithelial cytoplasm in an area of chronic progressive nephropathy; KO: KIM-1 globular staining is observed in proximal tubular epithelium (solid arrows) which colocalized with brown pigment noted on H&E sections. Scale bar = 0.5 mm

- not present; + present; ++ +++ ++++ indicate the relative number, size, and/or intensity of a finding as depicted in Figures 1-7.

A = H&E; B = Lipofuscin; C = CAB; D = LAMP-1; E = LAMP-2; F = NAGLU; WT/KO = KIM-1; NA = not applicable

### Histochemical stains

#### Kidney

Brown, granular-to-globular pigment was observed in the proximal tubular epithelium in the cortex (P1 and P2 segments) at 4-, 8-, and 12-months of age (Figures 4A, 5A, 6A) and the outer stripe (P3 segment) of the medulla of 8- and 12-months of age in LRRK2 KO rats. Pigment became more abundant and globular with age and distorted the cytoplasm, especially in the cortex. Renal medullary pigment also increased in abundance but was typically finely granular. The pigment was also demonstrated using a lipofuscin stain which highlighted the less abundant pigment noted at 4-months of age and the marked increase in pigment accumulation with age (Figures 4B, 5B, 6B). Lipofuscin positive pigment was not observed in 1- and 2-month old LRRK2 KO rats.

Hyaline droplets, characterized by eosinophilic, well-demarcated, intracytoplasmic globules, were observed in the proximal tubular epithelium at 2-, 4-, and 8-months of age in LRRK2 KO rats. Hyaline droplets in the proximal tubular epithelium of LRRK2 KO rats were minimally increased in number at 2-months of age. At 4-months of age, the droplets were more prominent, irregularly-shaped, larger and greater in number. Hyaline droplets were still observed at 8-months of age, but were less numerous, irregularly-shaped and smaller. Hyaline droplets were demonstrated using CAB stain (Figures 3C, 4C, 5C, 6C) which highlighted their shape, number, and distribution, and demonstrated their variable colocalization with intracytoplasmic brown pigment. Hyaline droplets were noted on CAB stained sections of kidney in the Long Evans WT rats started at 2-months of age but were pinpoint and represented normal intracytoplasmic protein (Figure 1C). Pigment accumulation and irregular hyaline droplets are more pronounced in the P1 and P2 segments of the proximal tubule (cortex) in the kidney of LRRK2 KO rats when compared to the P3 segment (medulla) and distal convoluted tubule (no LRRK2 changes observed). Phagocytosis and lysosomal activity along with sodium and chloride reabsorption are more extensive in the P1 and P2 segments. Alpha 2-U globulin, produced by the normal male rat liver, is phagocytosed and digested by the P1 and P2 segments. The small, regularly-shaped hyaline droplets in WT rats are consistent with normal phagocytic activity of this protein. The irregular hyaline droplets in LRRK2 KO rats suggest impaired lysosomal function since the irregular droplets are similar to those noted in alpha 2-U globulin nephropathy in male rats [24].

Cytoplasmic vacuolation of proximal tubular epithelium in the cortex was characterized by clear, well-delineated vacuoles in 4-, 8-, and 12-month old LRRK2 KO rats. Lesions consistent with chronic progressive nephropathy (CPN) characterized by basophilic tubules, thickened basement membranes and ± hyaline casts [24], were observed in Long Evans WT and LRRK2 KO rats at 4-, 8-, and 12-months of age. There was a slightly higher incidence of chronic progressive nephropathy in LRRK2 KO rats when compared to WT rats in the 4-month age groups but incidences and severities were similar at 8- and 12-months of age. The severities were minimal to mild at 4-months and minimal to moderate at 8- and 12-months of age (see Supplement S2 for grading scheme). LRRK2 KO-related microscopic findings were not observed in the distal convoluted tubules in any age group.

#### Liver, Lung, Heart, Spleen

The only LRRK2 KO-associated microscopic abnormality noted in 1-, 2-, 4-, 8- or 12-month old rats was minimal to mild centrilobular hepatocellular vacuolation in the liver of 2- and 8-month old rats in the second cohort. This vacuolation was not associated with hepatocellular degeneration. Vacuolation was not noted in the first cohort of rats, but these tissues were snap frozen and not formalin-perfused, which may have obscured hepatocellular vacuoles. There was no LRRK2 KO-related accumulation of pigment in these organs. LRRK2 KO-related abnormalities were not observed in the other tissues examined microscopically (see Supplement S4).

### Immunohistochemical stains of the Kidney

Lysosomes and some of their components were demonstrated using LAMP-1 (CD107a), LAMP-2 (CD107b), and n-acetylglucosaminidase (NAGLU) immunohistochemistry. In addition, Kidney Injury Molecule-1 (KIM-1), a marker of tubular epithelial injury, was assessed in the second cohort of rats.

#### WT LAMP-1 and LAMP-2 Expression

Baseline LAMP-1 and LAMP-2 expression in Long Evans WT kidneys was considered minimal at all ages. LAMP-1 staining of Long Evans WT kidneys was observed in the cortex (P1 and P2 segments) in proximal tubular epithelium and was characterized as diffuse, brown staining in the cytoplasm (Figure 1D). Baseline LAMP-2 staining of Long Evans WT kidneys was observed in the proximal tubules of the cortex (P1 and P2 segments) and outer stripe of the medulla (P3 segment). LAMP-2 staining was an intense brown in the apical portion of the cytoplasm adjacent to the tubular lumen (Figure 1E). 

#### LRRK2 KO LAMP-1 and LAMP-2 Expression

LAMP-1 expression in LRRK2 KO rat kidneys increased in intensity with age. In the 1- and 2-month age groups, LAMP-1 staining was minimal to mild in the cortex, diffuse in proximal tubular cytoplasm, and slightly increased in intensity when compared to the Long Evans WT group (Figures 2D, 3D). LAMP-1 medullary staining of 1- and 2-month-old LRRK2 KO rats was similar to the Long Evans WT group. LRRK2 KO rats at 4-, 8-, and 12-months displayed LAMP-1 staining that co-localized with brown pigment accumulation in the proximal tubular epithelium. In 4-month old LRRK2 KO rats, LAMP-1 staining was mild in the cortex, and minimal in the outer stripe of the medulla (Figure 4D). In 8- and 12-month-old LRRK2 KO rats, LAMP-1 staining was moderate and granular to globular in the cortex, and diffuse and mild in the medullary proximal tubular epithelium (Figures 5D, 6D). 

LAMP-2 expression in 1- and 2-month-old LRRK2 KO rats was similar to the Long Evans WT group (Figure 1E) and maintained the intense apical staining (Figures 2E, 3E). LAMP-2 staining was mild in 4-month-old LRRK2 KO rats (Figure 4E), and moderate in 8- and 12-month old LRRK2 KO rats (Figures 5E, 6E). LAMP-2 staining in the proximal tubules of the cortex was granular to globular and co-localized with brown pigment. As pigment accumulated with age, and LAMP-2 staining became more globular and increased in intensity, some apical staining persisted in proximal tubules. Medullary LAMP-2 staining in the outer stripe was mild and increased in intensity relative to the Long Evans WT group. 

#### WT NAGLU Expression

Baseline N-acetylglucosaminidase (NAGLU) expression in Long Evans WT kidneys was considered minimal at all ages. The positive staining was noted in the proximal tubular epithelium in the cortex and the outer stripe of the medulla. Positive staining was characterized by light brown, slightly granular staining in the cytoplasm. The second cohort of rats also demonstrated intense apical cytoplasmic NAGLU staining of proximal tubular epithelium of the cortex. Light brown, diffuse cytoplasmic staining was observed in the medulla. 

#### LRRK2 KO NAGLU Expression

NAGLU expression in 1-month-old LRRK2 KO rats (Figure 2F) was similar to the Long Evans WT groups (Figure 1F). In the 2-month old LRRK2 KO rats, the staining became slightly more globular in proximal tubules (Figure 3F). In 4-, 8-, and 12-month-old LRRK2 KO rats, NAGLU staining was progressively mild, moderate, and severe respectively (Figures 4F, 5F, 6F). The staining was characterized by variably-sized globules in the cortical proximal tubular epithelium and granular staining of the proximal tubule cytoplasm of the outer medullary stripe. NAGLU expression co-localized with observed brown pigment but not with hyaline droplets.

#### WT KIM-1 Expression

KIM-1 staining was examined in cohort 2 (1-, 2-, and 8-month old rats). Baseline KIM-1 staining of Long Evans WT rats was minimal and multifocal in 1- and 2-month-old rats and minimal to mild and multifocal in 8-month-old rats (Figure 7A). Positive KIM‑1 staining was dark brown and in the superficial cytoplasm and lumen of tubules in the cortex, medulla, and papilla. There was rare positive staining in the parietal epithelium of Bowman’s capsule. In 8-month-old WT rats, the mild staining was similar to that noted in 1- and 2-month-old rats with additional tubular staining in areas of chronic progressive nephropathy. 

#### LRRK2 KO KIM-1 Expression

KIM-1 staining in 1- and 2-month-old LRRK2 KO rats was similar to that of Long Evans WT rats. KIM-1 staining in the cortex of 8-month-old LRRK2 KO rats was characterized by dark brown variably-sized granules that co-localized with the brown pigment (Figure 7B). KIM-1 staining was occasionally intensely positive in the apical cytoplasm of pigment-laden cells. KIM-1 staining in the outer stripe was cytoplasmic, light brown, and granular. In addition, positive staining was noted in areas of chronic progressive nephropathy.

### Electron Microscopy (16-month old rats)

**Figure 8:**
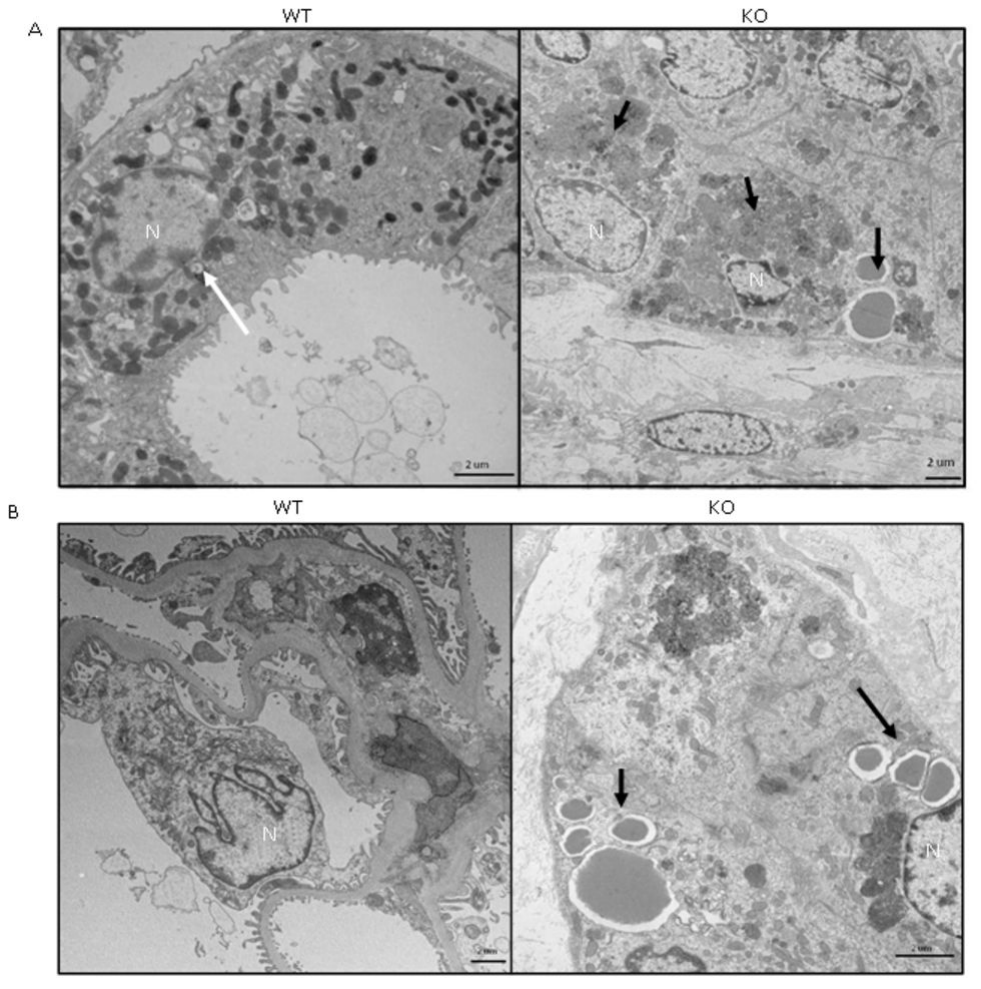
Morphological changes in the kidney of 16-month old LRRK2 KO rats vs. **WT**. (A) Increased number of lysosomes and electron dense material (black arrows) in KOs compared to WT (white arrow) (B) Accumulation of lipid droplets in glomerulus of KO animals compared to WT, which have no lipid droplets. N: Nucleus. Scale bar = 2 µm

The ultrastructure of 16-month old rats was examined since the greatest pathology might be seen in this older age group. The major morphological changes between the LRRK2 KO and WT animals were seen within the kidneys, lung, and liver. It was found that in the kidneys of the LRRK2 KO animals, there was an increase in the area and in the numbers of lysosomes in the proximal convoluted tubules when compared to the WT group (Figure 8). There were also further differences within the glomerulus, which had an accumulation of lipid droplets that were not seen in the WT animals. Other structures of the glomerulus and proximal convoluted tubules within the KO group were found to be morphologically similar to the WT animals. The distal convoluted tubules were found to be normal in the LRRK2 KO when compared to the wild types. 

**Figure 9:**
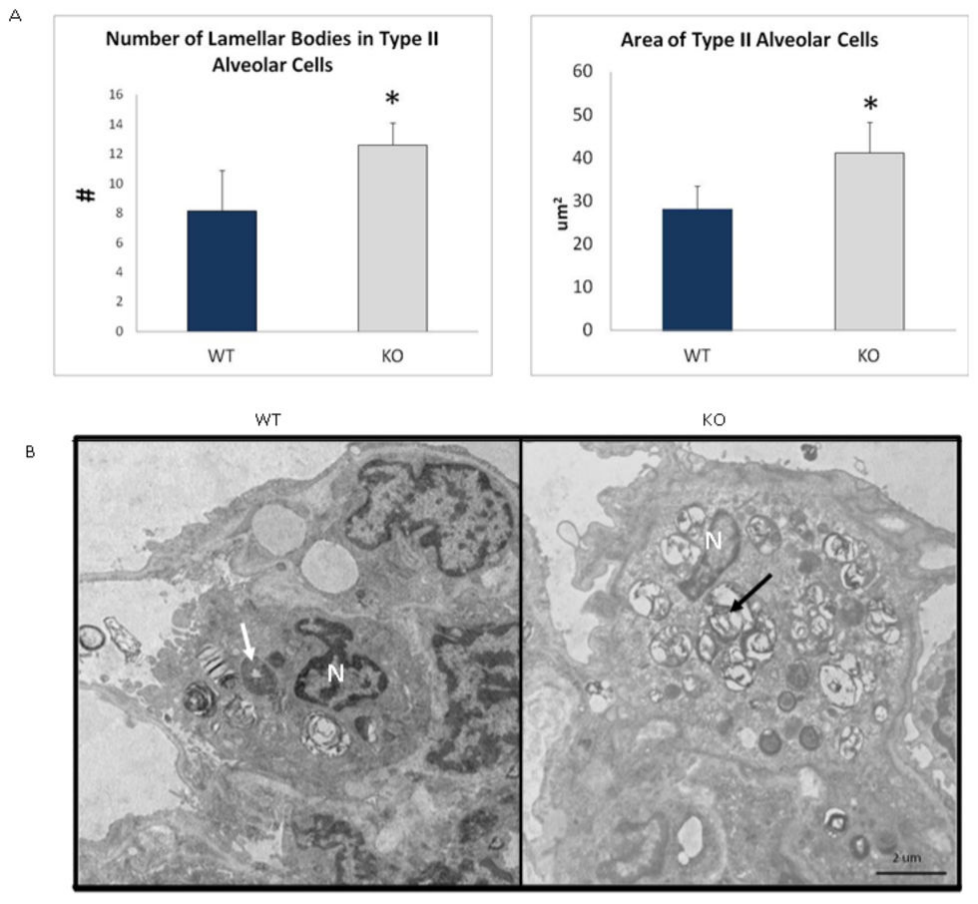
Accumulation of Lamellar Bodies in Type II Alveolar Cells in the lung of 16-month old LRRK2 KO rats vs. WT. (A) Graphs depicting Number of Lamellar Bodies in Type II Alveolar Cells and Area of Type II Alveolar Cells. (WT n=4, KO N=4) * - denote significance by Students *t*-test, P<0.05. Values are means + Standard Error of the Mean. (B) Normal Lamellar Body morphology (white arrow) in WT compared to proliferation of lamellar bodies (black arrow) in KO. N: Nucleus. Scale bar = 2 µm

Analysis of the lung revealed that Type II alveolar cells had significantly increased numbers of lamellar bodies, total area of lamellar bodies and area of Type II alveolar cells in the KO compared to WT animals (Figure 9). The average size of the lamellar bodies and density of lamellar bodies per cell were not significantly different (data not shown). The other cell components of the Type II alveolar cell as well as the Type I alveolar cell of the KO animals were found to be morphologically the same compared to the WT group.

**Figure 10:**
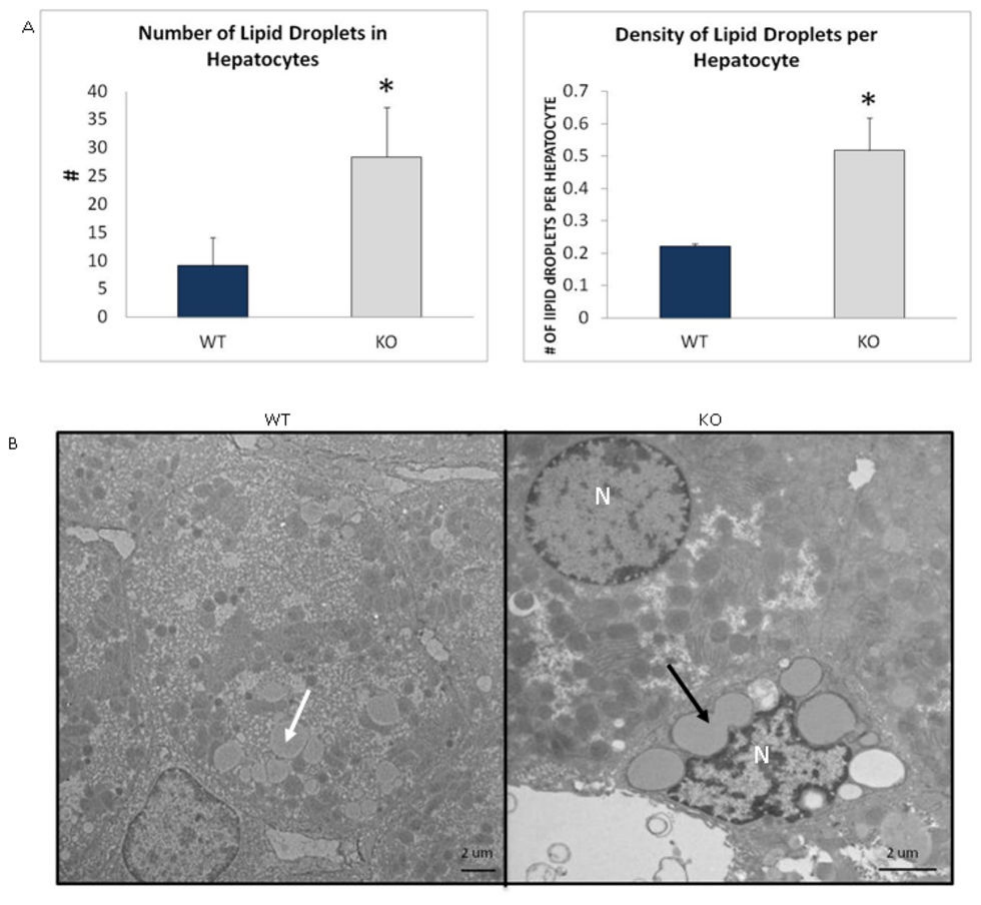
Accumulation of lipid droplets in hepatic cells in 16-month old LRRK2 KO rats vs. WT. (A) Graphs showing changes in number of lipid droplets and density of lipid droplets per hepatocyte. (WT n=2, KO n=4) *- denote significance by Student’s *t*-test, P<0.05. Values are means + Standard Error of the Mean. (B) Comparison of normal accumulation of lipid droplets (white arrow) in hepatic cells in WT to increased accumulation in hepatic cells (black arrow) in KOs. N: Nucleus. Scale bar = 2 µm

There was increased accumulation of lipid droplets in both hepatocytes and stellate cells of the KO compared to the WT animals (Figure 10). Densities of lipid droplets per cell were also found to be significantly increased in the KO rats when compared to the WT group (Figure 10). The area of the hepatic cells and total area of lipid droplets in each cell were not significantly different but showed a trend towards being increased in the KOs when compared to the WT group. There was no difference between the average areas of the lipid droplets between the two groups (data not shown). There were no other morphological changes found in the liver between the KO and WT animals. 

### Hematology and Coagulation (1-, 2-, and 8-month old rats)

LRRK2 KO-related hematological changes were minimal or occasionally mild and were mostly observed in all age groups (Table 2). There were lower red blood cell counts (all ages), lower hemoglobin values (1 month), lower hematocrit values (all ages), higher mean corpuscular volume (MCV; all ages), higher mean corpuscular hemoglobin (MCH; all ages) and mean corpuscular hemoglobin concentration (MCHC; all ages) values, higher hemoglobin distribution width (HDW) values (1- and 2-months) or lower HDW values (8-months), and lower red cell distribution width values (8-months). The percent and absolute reticulocyte counts were higher at 1- and 2-months of age but lower at 8-months of age. Mean platelet counts were higher at 1-, 2-, and 8-months of age.

**Table 2:** Summary of raw values and percent change of hematology and coagulation results for WT, 1-, 2-, and 8-month LRRK2 KO rats.

**Age / Strain**	**1 month**		**2 months**		**8 months**	
**Parameter Mean ± SEM**	**Long Evans WT**	**LRRK2 KO**	**Long Evans WT**	**LRRK2 KO**	**Long Evans WT**	**LRRK2 KO**
RBC (mil/µL)	7.12±0.065	6.47±0.136** (↓9.1%)	8.85±0.134	7.94±0.219* (↓10.3%)	9.60±0.164	8.98±0.093* (↓6.5%)
Hemoglobin(g/dL)	14.6±0.17	13.7±0.31* (↓6.2%)	16.5±0.10	16.3±0.35 (↓1.2%)	16.2±0.20	16.2±0.15 (0.00%)
Hematocrit (%)	45.7±0.71	42.1±1.10* (↓7.9%)	51.0±0.30	48.5±1.26 (↓4.9%)	50.3±0.67	48.6±0.30 (↓3.4%)
MCV (fL)	64.2±0.74	65.0±0.32 (↑1.3%)	57.7±0.53	61.0±0.15** (↑5.7%)	52.3±0.52	54.1±0.65 (↑3.4%)
MCH (pg)	20.5±0.25	21.1±0.12 (↑2.9%)	18.7±0.23	20.5±0.13** (↑9.6%)	16.9±0.23	18.1±0.26* (↑7.1%)
MCHC (g/dL)	32.0±.021	32.5±0.28 (↑1.6%)	32.4±0.15	33.7±0.17** (↑4.0%)	32.3±0.23	33.4*±0.13* (↑3.4%)
Absolute reticulocyte count (thous/µL)	267.7±17.98	365.3±17.56** (↑36.5%)	126.1±14.26	167.5±18.02 (↑32.8%)	217.2±23.45	141.0±5.19* (↓35.1%)
Reticulocytes (%)	3.8±0.25	5.7±0.21** (↑50.0%)	1.4±0.18	**2.1±0.28 (↑50.0%**)	2.3±0.26	1.6±0.06* (↓30.4)
RDW (%)	13.4±0.16	13.3±0.17 (↓0.8%)	11.0±0.05	11.0±0.30 (0.0%)	13.2±0.14	12.2±0.04** (↓7.6%)
HDW (g/dL)	1.59±0.036	1.90±0.025** (↑19.5%)	1.82±0.042	1.98±0.022* (↑8.8%)	2.55±0.100	2.16±0.058* (↓15.3%)
Platelets (thous/µL)	525±160.8	895±141.4 (↑70.5%)	692±33.8	888±29.1** (↑28.3%)	687±32.9	823±16.7* (↑19.8%)

* p<0.05; ** p<0.01; ↑ increase compared to WT control; ↓ decrease compared to WT control SEM Standard Error of the Mean

Differences from the Long Evans wild type group cited above were statistically significant (p<0.01 or p<0.05) except for hematocrit values at 2- and 8-months of age, MCV at 1- and 8- months of age, MCH at 1-month of age, MCHC at 1-month of age, platelet values at 1-month of age, and reticulocyte parameters at 2-months of age. While not all values were statistically significant, the trends for these values were similar in all three age groups with the exception of the percent and absolute reticulocyte values. The erythrocyte parameter changes were minimal to mild and suggested minimal to mild red blood cell loss with a reticulocyte response at 1- and 2-months of age. The reticulocyte response was not apparent at 8-months of age suggesting regenerative ability was not as robust. There was no microscopic bone marrow abnormalities associated with erythrocyte or platelet changes.

### Serum Chemistry (1-, 2-, and 8-month old rats)

LRRK2 KO-related higher cholesterol (all ages), higher phosphorous (2- and 8-months), lower chloride (all ages), lower sodium (2- and 8-months), and higher creatinine (8-months) were noted. Higher sorbitol dehydrogenase (SDH) was noted at 2- and 8-months of age (Table 3).

**Table 3:** Summary of serum chemistry results - Mean Differences (%) for LRRK2 KO rats compared to Long Evans WT.

**Age / Strain**	**1 month**		**2 months**		**8 months**	
**Parameter (Mean±SEM)**	**Long Evans WT**	**LRRK2 KO**	**Long Evans WT**	**LRRK2 KO**	**Long Evans WT**	**LRRK2 KO**
Cholesterol (mg/dL)	71±3.9	107±4.0** (↑50.7%)	57±3.3	83**±2.7 (↑45.6%)	56±7.0	94±9.8* (↑67.9%)
Creatinine (mg/dL)	0.17±0.013	0.18±0.009 (↑5.9%)	0.19±0.009	0.21±0.009 (↑10.5%)	0.28±0.015	0.34±0.013* (↑21.4%)
Phosphorous (mg/dL)	10.6±0.65	11.6±0.34 (↑9.4%)	8.4±0.03	10.0±0.26** (↑19.0%)	6.0±0.17	7.0±0.29* (↑16.7)
Chloride (mEq/L)	103±0.6	99±0.6** (↓3.9%)	105±0.5	102±0.5** (↓2.9%)	109±0.6	103±0.5** (↓5.5%)
Sodium (mEq/L)	144±0.9	143±0.6 (↓0.7%)	145±0.0	144±0.3* (↓0.7%)	148±0.3	144±0.3** (↓2.7%)
SDH (U/L)	9±.3.8	11±0.8 (↑22.2%)	5±0.6	12±1.6** (↑140%)	4±0.6	25±1.3** (↑525%)

* p<0.05; ** p<0.01; ↑ increase compared to WT control; ↓ decrease compared to WT control; SEM Standard Error of the Mean

### Urinalysis (1-, 2-, and 8-month old rats)

Urinalysis and urine chemistry values are summarized in Table 4. Urine specific gravity in LRRK2 KO rats was lower than Long Evans WT rats at 2-and 8-months of age and was associated with higher urine total volumes. Urine creatinine was lower and urine creatinine clearance was higher in LRRK2 KO rats but this effect was most pronounced at 2-months of age. Urine creatinine clearance was similar in 2- and 8-month LRRK2 KO rats. Urine sodium was higher and urine potassium was lower in LRRK2 KO rats with the change most pronounced in 2-month old rats. Urine chloride was lower in 1- and 2-month old LRRK2 KO rats and slightly higher in 8-month old rats. Urine electrolytes were normalized to urine creatinine, correcting for urine volume variability. Urine sodium/creatinine was higher in LRRK2 KO rats with a more pronounced change at 2-months of age. Urine potassium/creatinine values were elevated in 1-month old LRRK2 KO rats but similar to or slightly lower in 2- and 8-month LRRK2 KO rats. Urine chloride/creatinine values were higher in 1-, 2-, and 8-month LRRK2 KO rats with a more pronounced change at 2-months of age.

**Table 4:** Summary of urinalysis and urine chemistry for WT, 1-, -2, and -8-month old LRRK2 KO rats.

**Age / Strain**	**1 month**		**2 months**		**8 months**	
**Parameter (Mean±SEM)**	**Long Evans WT**	**LRRK2 KO**	**Long Evans WT**	**LRRK2 KO**	**Long Evans WT**	**LRRK2 KO**
Specific gravity	1.062±0.0017	1.062±0.0097 (0.0%)	1.032±0.0040	1.018±0.0005* (↓1.4%)	1.035±0.0015	1.026±0.0015** (↓0.9%)
Total volume (mL)	3.0±0.58	3.8±1.03 (↑26.7%)	3.5±0.65	13.3±1.03** (↑280%)	7.0±1.22	9.3±1.18 (↑32.8%)
Urine creatinine (mg/L)^b^	73.5±1.62	56.6±3.93* (↓23.0%)	69.4±7.83	38.0±3.26* (↓45.2%)	105.3±7.3	92.6±11.48 (↓12.1%)
Total urine creatinine clearance (mL/hr)^a^	88.39±14.76	98.07±4.89 (↑10.9%)	82.78±7.97	162.16±13.89** (↑95.9%)	169.29±15.96	162.94±17.74 (↓3.75%)
Urine sodium (mEq/L)	197±22.0	191±13.1 (↓3.0%)	38±12.4	45±2.6 (↑18.4%)	46±4.8	54±3.9 (↑17.4%)
Sodium/creatinine (mEq/mg)^c^	0.268±0.0280	0.337±0.0025 (↑25.7%)	0.052±0.0118	0.120±0.0093** (↑130.8%)	0.043±0.0034	0.062±0.005 (↑44.1%)
Urine potassium (mEq/L)	406.6±12.13	381.5±36.40 (↓6.2%)	188.5±20.65	105.2±1.87 (↓44.2%)	149.2±11.15	112.5±11.22 (↓24.6%)
Potassium/creatinine (mEq/mg)^c^	0.553±0.0057	0.672±0.0363* (↑21.5%)	0.273±0.0095	0.282±0.0209 (↑3.30%)	0.144±0.0154	0.125±0.0118 (↓13.2%)
Urine chloride (mEq/L)	320±19.7	305±26.2 (↓4.69%)	73±7.2	60±3.9 (↓17.8%)	66±5.1	70±5.7 (↑6.1%)
Chloride/creatinine (mEq/mg)^c^	0.435±0.0217	0.539±0.0312 (↑23.9%)	0.106±0.0066	0.158±0.0084** (↑49.0%)	0.065±0.0087	0.080±0.0138 (↑23.1%)

* p<0.05; ** p<0.01; ↑ increase compared to WT control; ↓ decrease compared to WT control; SEM Standard Error of the Mean

a = Creatinine clearance = urine concentration x (urine volume / collection period length [15 hours]) / serum concentration

b = urine creatinine measured as mg/dL and converted to mg/L for normalization of urine electrolytes

c = urine potassium, sodium and chloride (measured as mEq/L) were normalized to urine creatinine

## Discussion

The evidence is unequivocal that LRRK2 KO mice and rats exhibit an abnormal kidney phenotype [1,15–17]. We have replicated these findings but also found that these abnormalities in the LRRK2 KO rat progress with age, coincide with clinical pathology biomarkers, and extend to the lung and liver. Interestingly, the youngest LRRK2 KO cohort examined (1-month old), display clinical pathology that is not observed with gross examination, histochemical or immunohistochemical stains. Prior to the emergence of any abnormal phenotype, this cohort exhibits cholesterol, creatinine, phosphorous, chloride, sodium, and SDH alterations (see Table 3). Furthermore, the lung and liver of the oldest cohort examined (16-month old) display abnormal ultrastructure phenotypes that have never been previously reported. This highlights the importance in examining a wide-range of age groups and employing a myriad of techniques to uncover LRRK2 KO induced phenotypes. While we have replicated some of the morphological and clinical pathology findings found in the Ness et al. study [1] (e.g., increased body weight; altered cholesterol, red blood cell counts, and hematocrit percentage), extending the analysis to other age groups has further uncovered alterations in kidney, lung, and liver. It should be noted though that the liver findings appear to reflect more of a metabolic process abnormality than a lysosomal type observed in the kidney and lung since the EM liver results of increased lipid droplets are similar to the renal glomeruli phenotype. 

The identification of age-related phenotypes in the LRRK2 KO rats has important implications. First, it suggests that LRRK2 deficiency has deleterious effects over time that may first emerge prior to any gross morphological alterations. These early peripheral (e.g., blood or urine) signals may become safety biomarkers for future LRRK2 kinase inhibitor clinical trials. Secondly, it facilitates the selection of which LRRK2 KO rat aged cohorts to use for pharmacological mechanism-based safety studies. Examining potential on- or off-target effects of LRRK2 kinase inhibitors requires a LRRK2 KO animal model with a phenotype that will not mask potential safety liabilities of LRRK2 kinase inhibitors. The 1-month old LRRK2 KO cohort may be a better animal model than 2-, 4-, 8-, 12-, or 16-month old animals for LRRK2 kinase inhibitor safety experiments as it exhibits a milder phenotype with regards to gross morphology and histopathology. 

The challenge remains to ascertain the therapeutic window for a LRRK2 kinase inhibitor. It is important to note that all of the present studies were conducted using homozygous LRRK2 KO rats. Given that the LRRK2 heterozygous KO mouse kidney is devoid of kidney abnormalities and the lung abnormality is only associated with KO and not KD mice [16], pharmacological LRRK2 kinase inhibition of less than 50% may be tolerable. To ascertain this safety window, predictive safety and efficacy animal models are needed to determine the minimal amount of LRRK2 kinase inhibition that is required for the treatment of Parkinson’s disease. One challenge in developing a LRRK2 kinase inhibitor is that there is no robust *in vivo* model and only a few pharmacodynamic (PD) readouts (e.g., pSer935 and pSer1292) that can be used to screen the efficacy of potential LRRK2 kinase inhibitors. Without knowing the minimal LRRK2 kinase inhibition that is required to obtain efficacy, a therapeutic index is unobtainable. 

It is plausible, however, that genetically induced abnormal phenotypes in rodents may not translate to other species (e.g., dog, non-human primates, and humans) and/or be predictive of LRRK2 pharmacological induced toxicity. For example, the elevated cholesterol observed in the LRRK2 KO rats is not a good model of human cholesterol related diseases such as atherosclerosis [25] since rat serum cholesterol is primarily composed of high density lipoproteins. Also, elevated SDH (a marker of liver damage) observed in LRRK2 KO rats was not associated with hepatocellular degeneration. SDH is expressed in the kidney, but typically has its highest expression in the regions of the kidney that did not show any abnormalities in the LRRK2 KO rats (i.e., glomeruli and distal convoluted tubules) [26]. Therefore, future experiments need to determine the implications of chronic exposure to potent and selective LRRK2 kinase inhibitors on both rodent and non-rodent species.

If these pathological observations are related to LRRK2 kinase inhibition in non-rodent species and are predictive of clinical pathology then the identification of cerebral spinal fluid (CSF) biomarkers along with peripheral (e.g., blood and/or urine) safety markers could be crucial for the development of LRRK2 kinase inhibitors. One recent promising PD approach is to measure LRRK2 released from exosomes in the CSF and urine [27]. The prediction is that LRRK2 kinase inhibitors will diminish the total LRRK2 levels secreted into exosomes and allow for the measurement of LRRK2 target engagement from accessible sampling compartments [27]. A CSF biomarker would especially facilitate a first-in-class LRRK2 kinase inhibitor human trial by allowing the clinician to monitor the relationship between brain LRRK2 kinase activity and safety.

Although LRRK2 genetic rodent evidence suggests potential issues in inhibiting LRRK2 kinase activity, it is important to note that none of the LRRK2 KO induced phenotypes reported to date translate to detrimental functional deficits [1,15–17]. Further pre-clinical studies examining pharmacological inhibition of kinase activity in non-rodent species and the identification of safety/efficacy biomarkers are needed. With pharmaceutical companies making advances in developing LRRK2 kinase inhibitors, it is crucial that we exhaust all means to bring a safe drug into the clinic. 

## Supporting Information

Supplement S1 **Histochemical and immunohistochemical staining procedures.**
(DOC)

Supplement S2 **Grading of histochemical and immunohistochemical stain sections.**
(DOCX)

Supplement S3 **Clinical pathology parameters.**
(DOCX)

Supplement S4 **Macroscopic and microscopic examination.**
(DOCX)
