# Supplementary material for: Loss of Leucine-Rich Repeat Kinase 2 (LRRK2) in Rats Leads to Progressive Abnormal Phenotypes in Peripheral Organs
Source: PLoS One. 2013 Nov 14;8(11):e80705. doi: 10.1371/journal.pone.0080705 (PMC3828242; doi:10.1371/journal.pone.0080705)
Supplement: Supplement S1 — Histochemical and immunohistochemical staining procedures. (DOC) [file pone.0080705.s001.doc]

**Supplement S1 -** Histochemical and Immunohistochemical Staining Procedures

| **AFIP Method for Lipofuscin Staining** | |
| --- | --- |
| Step | Procedure |
| 1 | Deparaffinize and hydrate sections to distilled water. |
| 2 | Stain in filtered Kinyoun's carbol fuchsin solution for 1 hour. |
| 3 | Rinse in distilled water. |
| 4 | Differentiate with 1% acid alcohol until sections are pale pink - 5-6 dips. |
| 5 | Wash thoroughly in running water for 5 minutes, then rinse in distilled water. |
| 6 | Counterstain in picric acid solution until slides appear yellow - 5-8 dips. |
| 7 | Dehydrate 95% and 100% alcohols. Clear in xylene and coverslip. |
|  | **Reagents:**  Kinyoun's Carbol Fuchsin  Phenol, liquid 8.0mL  95% alcohol 20mL  Basic fuchsin 4 gm.  Deionized water 1OOmL  Dissolve basic fuchsin in the alcohol and phenol in the distilled water. Mix the two solutions. Filter.  1% Acid Alcohol  70% Alcohol 990mL  Hydrochloric acid, concentrated 1OmL  Picric Acid Solution  Picric Acid, approximately 1.18gm.  Distilled water 1OOmL |

| **Chromotrope-Aniline Blue (CAB) Staining Procedure [19]** | |
| --- | --- |
| Step | Procedure |
| 1 | Deparaffinize and hydrate to distilled water. |
| 2 | Stain in Weigert’s hematoxylin - 2 minutes |
| 3 | Rinse in distilled water. |
| 5 | Place in 1% Phosphomolybdic acid - 2 minutes. |
| 6 | Rinse slide in distilled water. |
| 7 | Stain in CAB solution - 4 minutes. |
| 8 | Rinse slide in distilled water. |
| 9 | Dehydrate quickly with 2 changes of 100% alcohol. |
| 10 | Clear with 2 changes of xylene (or substitute) and coverslip. |
|  | **CAB solution**  Hydrochloric acid - 2.5mL  Distilled water - 200mL  Aniline blue - 1.5g  Mix with gentle heat, then add 6.0g Chromotrope 2R. Filter prior to use. |

| **LAMP-1 Immunohistochemistry Staining Procedure** | |
| --- | --- |
| Step | Procedure on Ventana Discovery XT, Protocol #186 |
| 1 | Warm slides to 75ºC. |
| 2 | Deparaffinize with EZ Prep (Ventana, Tucson, AZ, reference no. 950-100). |
| 3 | Apply Liquid Coverslip (Ventana, Tucson, AZ, reference no. 650-010). |
| 4 | Antigen retrieval with Standard CC1Mild incubation at 95°C (Cell Conditioning 1, EDTA pH 8.0) (Ventana, Tucson, AZ, reference no. 950-124). |
| 5 | Rinse slide with Reaction Buffer (Ventana, Tucson, AZ, reference no. 950-300). |
| 6 | Apply Liquid Coverslip. |
| 7 | Apply Inhibitor CM and incubate for 4 minutes (Ventana, Tucson, AZ, reference no. 760‑159). |
| 8 | Rinse slide with Reaction Buffer. |
| 9 | Apply primary antibody anti-LAMP-1 for 28 minutes (Santa Cruz Biotechnology, reference no.sc-17768). |
| 10 | Rinse slide with Reaction Buffer. |
| 11 | Apply OmniMap anti-Mouse HRP and incubate for 16 minutes (Ventana, Tucson, AZ, reference no. 760-4310). |
| 12 | Rinse slide with Reaction Buffer. |
| 13 | Apply DAB CM and DAB H_2_O_2_ CM and incubate for 8 minutes (Ventana, Tucson, AZ, reference no. 760-159). |
| 14 | Rinse slide with Reaction Buffer. |
| 15 | Apply Copper CM and incubate for 4 minutes (Ventana, Tucson, AZ, reference no. 760‑159). |
| 16 | Rinse slide with Reaction Buffer. |
| 17 | Apply Hematoxylin and incubate for 4 minutes. (Ventana, Tucson, AZ, reference no. 760‑2021). |
| 18 | Rinse slide with Reaction Buffer. |
| 19 | Apply Bluing Reagent and incubate for 4 minutes (Ventana, Tucson, AZ, reference no. 760-2037). |
| 20 | Rinse slide with Reaction Buffer. |
| 21 | Remove slides, dehydrate, and coverslip. |

#

| **LAMP-2 Immunohistochemistry Staining Procedure** | |
| --- | --- |
| Step | Procedure on Ventana Discovery XT, Protocol #190 |
| 1 | Warm slides to 75ºC. |
| 2 | Deparaffinize with EZ Prep (Ventana, Tucson, AZ, reference no. 950-100). |
| 3 | Apply Liquid Coverslip (Ventana, Tucson, AZ, reference no. 650-010). |
| 4 | Antigen retrieval with Standard CC1 incubation at 95°C (Cell Conditioning 1, EDTA pH 8.0) (Ventana, Tucson, AZ, reference no. 950-124). |
| 5 | Rinse slide with Reaction Buffer (Ventana, Tucson, AZ, reference no. 950-300). |
| 6 | Apply Liquid Coverslip. |
| 7 | Apply Inhibitor CM and incubate for 4 minutes (Ventana, Tucson, AZ, reference no. 760‑159). |
| 8 | Rinse slide with Reaction Buffer. |
| 9 | Apply primary antibody anti-LAMP-2 for 20 minutes (Santa Cruz Biotechnology, reference no.sc-5571). |
| 10 | Rinse slide with Reaction Buffer. |
| 11 | Apply OmniMap anti-Rabbit HRP and incubate for 12 minutes (Ventana, Tucson, AZ, reference no. 760-4311). |
| 12 | Rinse slide with Reaction Buffer. |
| 13 | Apply DAB CM and DAB H_2_O_2_ CM and incubate for 8 minutes (Ventana, Tucson, AZ, reference no. 760-159). |
| 14 | Rinse slide with Reaction Buffer. |
| 15 | Apply Copper CM and incubate for 4 minutes (Ventana, Tucson, AZ, reference no. 760‑159). |
| 16 | Rinse slide with Reaction Buffer. |
| 17 | Apply Hematoxylin and incubate for 4 minutes. (Ventana, Tucson, AZ, reference no. 760‑2021). |
| 18 | Rinse slide with Reaction Buffer. |
| 19 | Apply Bluing Reagent and incubate for 4 minutes (Ventana, Tucson, AZ, reference no. 760-2037). |
| 20 | Rinse slide with Reaction Buffer. |
| 21 | Remove slides, dehydrate, and coverslip. |

| **NAGLU Immunohistochemistry Staining Procedure** | |
| --- | --- |
| Step | Procedure on Ventana Discovery XT, Protocol #219 |
| 1 | Warm slides to 75ºC. |
| 2 | Deparaffinize with EZ Prep (Ventana, Tucson, AZ, reference no. 950-100). |
| 3 | Apply Liquid Coverslip (Ventana, Tucson, AZ, reference no. 650-010). |
| 4 | Antigen retrieval with Standard CC1Standard incubation at 95°C (Cell Conditioning 1, EDTA pH 8.0) (Ventana, Tucson, AZ, reference no. 950-124). |
| 5 | Rinse slide with Reaction Buffer (Ventana, Tucson, AZ, reference no. 950-300). |
| 6 | Apply Liquid Coverslip. |
| 7 | Apply Inhibitor CM and incubate for 4 minutes (Ventana, Tucson, AZ, reference no. 760‑159). |
| 8 | Rinse slide with Reaction Buffer. |
| 9 | Apply option, background sniper for 8 minutes (Biocare, Concord, CA, reference no. NBP1-82601 |
| 10 | Apply primary antibody anti-NAGLU for 28 minutes (Novus Biologicals, reference no.NBP1-82601). |
| 11 | Rinse slide with Reaction Buffer. |
| 12 | Apply OmniMap anti-Rabbit HRP and incubate for 20 minutes (Ventana, Tucson, AZ, reference no. 760-4311). |
| 13 | Rinse slide with Reaction Buffer. |
| 14 | Apply DAB CM and DAB H_2_O_2_ CM and incubate for 8 minutes (Ventana, Tucson, AZ, reference no. 760-159). |
| 15 | Rinse slide with Reaction Buffer. |
| 16 | Apply Copper CM and incubate for 4 minutes (Ventana, Tucson, AZ, reference no. 760‑159). |
| 17 | Rinse slide with Reaction Buffer. |
| 18 | Apply Hematoxylin and incubate for 4 minutes. (Ventana, Tucson, AZ, reference no. 760‑2021). |
| 19 | Rinse slide with Reaction Buffer. |
| 20 | Apply Bluing Reagent and incubate for 4 minutes (Ventana, Tucson, AZ, reference no. 760-2037). |
| 21 | Rinse slide with Reaction Buffer. |
| 22 | Remove slides, dehydrate, and coverslip. |

| **KIM-1 Immunohistochemistry Staining Procedure** | |
| --- | --- |
| Step | Procedure on Ventana Discovery XT, Protocol #240 |
| 1 | Warm slides to 75ºC. |
| 2 | Deparaffinize with EZ Prep (Ventana, Tucson, AZ, reference no. 950-100). |
| 3 | Apply Liquid Coverslip (Ventana, Tucson, AZ, reference no. 650-010). |
| 4 | Antigen retrieval with Standard CC2Mild incubation at 95°C (Cell Conditioning 1, EDTA pH 8.0) (Ventana, Tucson, AZ, reference no. 950-124). |
| 5 | Rinse slide with Reaction Buffer (Ventana, Tucson, AZ, reference no. 950-300). |
| 6 | Apply Liquid Coverslip. |
| 7 | Apply Inhibitor CM and incubate for 4 minutes (Ventana, Tucson, AZ, reference no. 760‑159). |
| 8 | Rinse slide with Reaction Buffer. |
| 9 | Apply primary antibody anti-KIM-1 for 24 minutes (R&D Systems, reference no.AF3689). |
| 10 | Rinse slide with Reaction Buffer. |
| 11 | Apply OmniMap anti-Goat HRP and incubate for 16 minutes (Ventana, Tucson, AZ, reference no.760-4647). |
| 12 | Rinse slide with Reaction Buffer. |
| 13 | Apply DAB CM and DAB H_2_O_2_ CM and incubate for 8 minutes (Ventana, Tucson, AZ, reference no. 760-159). |
| 14 | Rinse slide with Reaction Buffer. |
| 15 | Apply Copper CM and incubate for 4 minutes (Ventana, Tucson, AZ, reference no. 760‑159). |
| 16 | Rinse slide with Reaction Buffer. |
| 17 | Apply Hematoxylin and incubate for 4 minutes. (Ventana, Tucson, AZ, reference no. 760‑2021). |
| 18 | Rinse slide with Reaction Buffer. |
| 19 | Apply Bluing Reagent and incubate for 4 minutes (Ventana, Tucson, AZ, reference no. 760-2037). |
| 20 | Rinse slide with Reaction Buffer. |
| 21 | Remove slides, dehydrate, and coverslip. |
