# Supplementary material for: Loss of Leucine-Rich Repeat Kinase 2 (LRRK2) in Rats Leads to Progressive Abnormal Phenotypes in Peripheral Organs
Source: PLoS One. 2013 Nov 14;8(11):e80705. doi: 10.1371/journal.pone.0080705 (PMC3828242; doi:10.1371/journal.pone.0080705)
Supplement: Supplement S2 — Grading of histochemical and immunohistochemical stain sections. (DOCX) [file pone.0080705.s002.docx]

| Grade 1 (Minimal) | A histologic change that barely exceeds that which is considered to be within normal limits. This change is unlikely to produce any functional impairment or is normal background of histochemical or immunohistochemical marker or minimal increase in staining. |
| --- | --- |
| Grade 2 (Mild) | A histologic change that is easily identified but of limited severity. This change probably does not produce any functional impairment or represents a minimal to mild increase in staining intensity and/or change in staining pattern. |
| Grade 3 (Moderate) | A histologic change that is a prominent feature of the tissue but not of maximal severity. Limited tissue or organ dysfunction is possible or represents a moderate increase in staining intensity and/or change in staining pattern. |
| Grade 4 (Severe) | A histologic change that is an overwhelming feature of the tissue and that may cause significant tissue or organ dysfunction or represents a severe increase in staining intensity and/or change in staining pattern. |

**Supplement S2-** Grading of histochemical and immunohistochemical stain sections.
