# Supplementary material for: Loss of Leucine-Rich Repeat Kinase 2 (LRRK2) in Rats Leads to Progressive Abnormal Phenotypes in Peripheral Organs
Source: PLoS One. 2013 Nov 14;8(11):e80705. doi: 10.1371/journal.pone.0080705 (PMC3828242; doi:10.1371/journal.pone.0080705)
Supplement: Supplement S3 — Clinical pathology parameters. (DOCX) [file pone.0080705.s003.docx]

**Supplement S3-** Clinical pathology parameters

Hematology and Coagulation

| Total leukocyte count (WBC)  Erythrocyte count (RBC)  Hemoglobin (HGB)  Hematocrit (HCT)  Mean corpuscular volume (MCV)  Mean corpuscular hemoglobin (MCH)  Mean corpuscular hemoglobin concentration (MCHC)  Platelet count (Platelet)  Prothrombin time (PT)  Activated partial thromboplastin time (APTT)  Reticulocyte count  Percent (Retic)  Absolute (Retic Absolute) | Mean platelet volume (MPV)  Red cell distribution width (RDW)  Hemoglobin distribution width (HDW)  Differential leukocyte count -  Percent and absolute  -Neutrophil (NEU)  -Lymphocyte (LYMPH)  -Monocyte (MONO)  -Eosinophil (EOS)  -Basophil (BASO)  -Large unstained cell (LUC)  Platelet estimate a  Red cell morphology (RBC Morphology) a |
| --- | --- |

( ) = Designates abbreviations used in data tables

a = Presented on individual tables if a manual differential was performed, and the manual data were accepted and reported instead of the automated differential data

Serum Chemistry

| Albumin  Total protein  Globulin [by calculation]  Albumin/globulin ratio (A/G Ratio) [by calculation]  Total bilirubin (Total Bili)  Urea nitrogen  Creatinine  Alkaline phosphatase (ALP)  Alanine aminotransferase (ALT)  Aspartate aminotransferase (AST) | Gamma glutamyltransferase (GGT)  Glucose  Total cholesterol (Cholesterol)  Calcium  Chloride  Phosphorus  Potassium  Sodium  Triglycerides (Triglyceride)  Sorbitol dehydrogenase (SDH)  Appearance a |
| --- | --- |

( ) = Designates abbreviation used in data tables

a = Includes the degree of hemolysis, icterus, and lipemia (presented on individual data tables only).

Urinalysis

| Specific gravity (SG)  pH  Urobilinogen (URO)  Total volume (TVOL)  Color (COL)  Clarity (CLA)  Protein (PRO)  Glucose (GLU)  Ketones (KET)  Bilirubin (BIL) | Occult blood (BLD)  Leukocytes (LEU)  Nitrites (NIT)  Microscopy of sediment [Tabular abbreviations appear on individual tables]  Sodium^a^  Potassium^a^  Chloride^a^  Creatinine^a^ |
| --- | --- |

( ) = Designates abbreviations used in data tables

a = Calculated urine chemistry parameters are reported
