# Supplementary material for: Loss of Leucine-Rich Repeat Kinase 2 (LRRK2) in Rats Leads to Progressive Abnormal Phenotypes in Peripheral Organs
Source: PLoS One. 2013 Nov 14;8(11):e80705. doi: 10.1371/journal.pone.0080705 (PMC3828242; doi:10.1371/journal.pone.0080705)
Supplement: Supplement S4 — Macroscopic and microscopic examination. (DOCX) [file pone.0080705.s004.docx]

**Supplement S4-** Macroscopic and Microscopic Examination

| Adrenal glands (2)*  Aorta*  Bone with marrow (sternum, femur)  Bone marrow smear (from femur)^a^  Brain† (cerebrum, cerebellum*,   striatum [left and right]*)  Epididymides (2)^b^  Eyes with optic nerve (2)^b^*  Gastrointestinal tract  Esophagus*  Stomach*  Duodenum*  Jejunum*  Ileum*  Cecum*  Colon*  Rectum*  Heart+*  Kidneys (2)+*  Larynx  Liver (sections of 2 lobes)+*  Lungs (including bronchi)+*  Lymph node  Axillary (2)*  Mandibular (2)  Mesenteric* | Pancreas*  Peripheral nerve (sciatic)  Peyer’s patches  Pharynx  Pituitary*  Prostate*  Salivary glands [mandibular (2)]  Seminal vesicles (2)  Skeletal muscle (rectus femoris)*  Skin (with section taken from same  anatomic area as female mammary  gland)*  Spinal cord (cervical*, thoracic,   lumbar)  Spleen+*  Testes (2)^b^  Thymus*  Thyroids [with parathyroids (2)]^c^*  Tongue  Trachea  Urinary bladder  All gross lesions (per SOP) |
| --- | --- |

Note: Unless otherwise noted, all listed organs were examined both macroscopically and microscopically

^a^ = Not taken from animals found dead; not placed in formalin. Only examined if scientifically warranted (based on hematology and histopathologic findings).

^b^ = Testes and epididymides placed in modified Davidson’s solution; eyes and optic nerve placed in Davidson’s solution.

^c^ = Parathyroids examined if present in the plane of section and in all cases where a gross lesion was present.

* = Organ weighed for all animals in the first cohort (4-, 8-, and 12-months of age)

† = Organ weighed and measured (length and width) for all animals

+ = Additional section(s) of this organ examined microscopically following special (histochemical and/or immunohistochemical) staining procedures.
